# Laser-Induced-Plasma-Assisted Ablation and Metallization on C-Plane Single Crystal Sapphire (c-Al_2_O_3_)

**DOI:** 10.3390/mi8100300

**Published:** 2017-10-07

**Authors:** Xizhao Lu, Feng Jiang, Tingping Lei, Rui Zhou, Chentao Zhang, Gaofeng Zheng, Qiuling Wen, Zhong Chen

**Affiliations:** 1 College of Mechanical Engineering and Automation, Huaqiao University, Xiamen 361021, China; jiangfeng@hqu.edu.cn (F.J.); ltp9851@xmu.edu.cn (T.L.); qlwen@hqu.edu.cn (Q.W.); 2 Department of Electronic Science, Xiamen University, Xiamen 361005, China; 3 Fujian Key Laboratory of Special Energy Manufacturing, Huaqiao University, Xiamen 361021, China; 4 School of Aerospace Engineering, Xiamen University, Xiamen 361005, China; rzhou2@xmu.edu.cn (R.Z.); zctyyy@163.com (C.Z.); zheng_gf@xmu.edu.cn (G.Z.)

**Keywords:** single crystal sapphire, laser-induced-plasma-assisted ablation, repetition rate, metallization, micro-machines

## Abstract

Laser-induced-plasma-assisted ablation (LIPAA) is a promising micro-machining method that can fabricate microstructure on hard and transparent double-polished single crystal sapphire (SCS). While ablating, a nanosecond pulse 1064 nm wavelength laser beam travels through the SCS substrate and bombards the copper target lined up behind the substrate, which excites the ablating plasma. When laser fluence rises and is above the machining threshold of copper but below that of SCS, the kinetic energy of the copper plasma generated from the bombardment is mainly determined by the laser fluence, the repetition rate, and the substrate-to-target distance. With a lower repetition rate, SCS becomes metallized and gains conductivity. When micro-machining SCS with a pulsed laser are controlled by properly controlling laser machining parameters, such as laser fluence, repetition rate, and substrate-to-target distance, LIPAA can ablate certain line widths and depths of the microstructure as well as the resistance of SCS. On the contrary, conductivity resistance of metalized sapphire depends on laser parameters and distance in addition to lower repetition rate.

## 1. Introduction

Single crystal sapphire (SCS) is a hard and transparent material widely used in various fields such as optics, LED, and optoelectronics. It is a great challenge to produce consistent precision and a conductive microstructure with a pulsed laser on a surface with high transparency, high strength, high rigidity, and abrasion resistance. Laser-induced-plasma-assisted ablation (LIPAA) [1,2,3] is a method that can ablate the microstructure on sapphire, diamond, and other, similar materials. The lattice constant of c-plane SCS is much higher than that of a-plane SCS, respectively; *a* = 4.78 and *c* = 12.99. That is to say, the cell of the c-plane is much larger than that of the alpha plane sapphire, and sapphire shows a high anisotropic behavior; as a result, the ablating efficiency of LIPAA on c-plane sapphire is different from that on a-plane sapphire. The energy range of a-plane (2$\bar{1}\bar{1}$0) sapphire substrates is 0.75–6.5 eV at room temperature [4,5], which obviously differs from those of the c-plane (1110) [6,7,8]. C-plane SCS has excellent characteristics, including chemical stability, a high dielectric constant, high hardness (with a Mosh scale hardness level of 9), and moderate heat dispersion [9,10]. It demonstrates sound diaphaneity with regard to 1064 nm wavelength lasers. In fact, the preferable absorption wavelength of sapphire is about 200 nm [11,12,13]. There are several methods of processing SCS, such as ultrasonic assisted grinding process [14], UV laser cutting, micro-laser assisted machining [15,16], and LIPAA.

LIPAA, a versatile method, can be used to fabricate desired microstructures for heaters, guide-wave devices, and LED substrates. In 2004, Yasutaka Hanada and Koji Sugioka worked on ablation sapphire research on the c-plane [17].They focused on the sapphire ablation, but they did not study metallization and the lattice structure of material. In this work, we use plasma induced by nanosecond or femtosecond laser pulses for precision machining development of microstructures on SCS surfaces. The difference in the ablation line width and the depth lies between c-plane and a-plane SCS because of their different lattices. The nanosecond laser pulse focuses on the copper target behind the transparent SCS, which induces plasma that bombards the surface. The SCS nanosecond/femtosecond pulse duration laser is used precisely to make LIPAA ablate microstructures on hard and transparent sapphire [18,19].

## 2. Setup and Experiment

**Figure 1:**
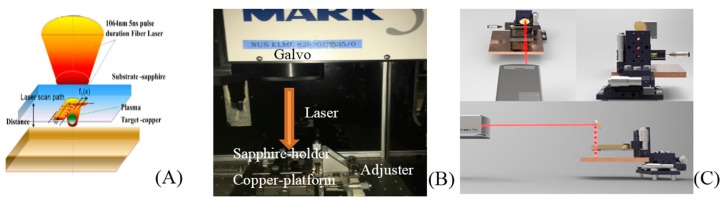
(**A**) Schematic diagram of LIPAA working principle. (**B**) Setup of the LIPAA platform. (**C**) 3D view of LIPAA on a SCS surface.

Figure 1A shows the working principle of LIPAA on SCS. The copper target lies beneath the transparent substrate. Laser beams go through the sapphire and focus on the copper target. Figure 1B shows the setup of an adjustable stage (the minimal adjustable distance is 10 μm) used in the experiment. The time-of-flight (TOF) spectra of the plasma are considered to be a shifted Maxwell–Boltzmann distribution, which is related to the substrate-to-target distance and ablation overlap rate; that is to say, it is related to the flight distance as well as the initial velocity and energy of the excited plasma.

A 1064 nm wavelength fiber laser (average power 18 W, 1064 nm wavelength fiber laser MARK5, IPG series Model: YLPR-0.3-A1-60-18) is used as the energy source of LIPAA. The range of adjustable repetition rate is 10–300 kHz, and the pulse width can be set to 1, 5 or 10 ns. The maximum energy of a single pulse is 200–300 μJ. C-plane SCS substrates used in the experiment were provided by Shanghai Duopu Optics Materials Company. The flat copper targets were placed behind the sapphire substrate mounted on an adjustable bracket (with an adjustable range of 50–1700 μm). When the ablating experiments begin, the sapphire substrate and the copper target immersed in the acetone solution are cleaned with an ultrasonic cleaning machine, rinsed with deionized water via ultrasonic cleaning, and dried with nitrogen.

While the surfaces of the copper target are heated by nanosecond laser pulses, it emits bright green light due to a copper flame reaction; that is to say, the laser beam passes through the substrate and hits the copper target, exciting the plasma. The exciting energy of the laser beam has a significant impact on the ablating process. It is obvious that, in the initial stage, within a certain range, the higher the energy of the laser beam is, the higher the velocity and energy of the excited plasma will be. However, when the laser energy exceeds the demand of the exciting copper ions, the outer layer copper ablated and the copper ion ablation occurs. The large amount of copper deposits on the outer layer obstructs the acceleration of newly excited copper ions; consequently, the ablation line width and depth decrease.

When laser fluence reaches the ablating threshold, adjusting the repetition rate becomes critical. Under the same fluence, by adjusting the substrate-to-target distance from 50 to 1700 μm, the plasma TOF is changed. The longer the distance is, the less controlled the plasma is, and the weaker the linearity with respect to the impact of the Maxwell–Boltzmann distribution.

To reduce the risk of micro-thermal cracks on SCS, the pulse width is often limited to 1 ns or 5 ns. In the initial stage, the laser fluence is lower than the ablating threshold of SCS, so laser beams do not directly ablate the surface of SCS; instead, they travel through SCS and excite the plasma, which in turn bombards SCS on the alumina surface or even on the covalent bonds. This is the deposition or ablation process on SCS. Increased beam energy means increased plasma kinetic energy. A higher overlapping rate indicates that many copper ions are participating in the ablation or deposition. A lower repetition rate indicates a lower overlapping rate, a substrate-to-target distance affect ablation micro structure profiles as well.

## 3. Results and Discussion

In the process of LIPAA, SCS can be considered a high thermal capability material because of its limited thermal expansion coefficient (about 28 W·m^−1^·K^−1^ in proportion to the volume of sapphire samples [20]). The thermal capability of c-plane SCS is higher than that of a-plane SCS, and the ablating threshold of the two is different. In fact, it is difficult to ablate with a short pulse laser. For instance, in the brittle SCS, micro-cracks may develop due to the microstructure or high thermal sensitivity. After all, high hardness, high thermal sensitivity, and the chemical stability are the main obstacles to microstructure ablation on SCS.

During the LIPAA process, the substrate-to-target distance and laser fluence play major roles. According to Planckian radiation law, with a shorter wavelength, a shorter pulse duration, and high repetition rate, photons of a laser beam will have higher energy, and the excited plasma is more capable of ablating the SCS; conversely, copper ions are deposited on SCS and become conductive.

**Figure 2:**
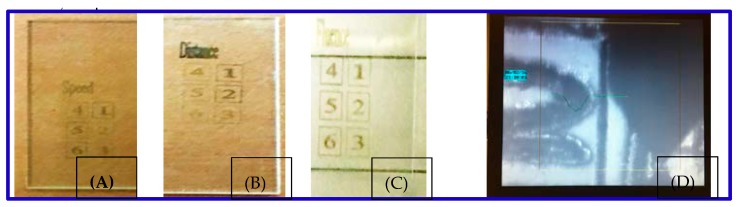
C-plane sapphire samples of LIPAA. (**A**) Impact of laser scanning speed on line width and depth. (**B**) Impact of substrate-to-target distance on line width and depth. (**C**) Impact of laser fluence on line width and depth. (**D**) One of profiles measured with the stylus profilometer.

Figure 2A shows samples where the laser scan speed affects the microstructure on the sapphire, when the plasma fluence and overlapping increase, which is tuned above the plasma breakdown threshold of SCS, and the substrate-to-target distance are fixed. Both the ablation depth and width increase with higher laser overlapping, which corresponds to a lower scan speed. Figure 2B shows the impact of substrate-to-target distance in LIPAA when the fluence and scanning speed are fixed. Figure 2C shows the impact of fluence on the ablation of SCS. Figure 2D shows the impact on SCS metallization from the parameters of the scan speed, distance, and fluence, measured with the stylus profilometer.

Attributing to its high sensitivity and simplicity, the LIPAA method can modify nonlinear characteristics of optics materials. With traditional processing methods, the 1064 nm nanosecond laser cannot ablate SCS directly. There are two major factors, namely, the substrate-to-target distance and the interval between two atoms that determine the ablating or depositing processes occurring on the rear-side of the SCS. To form a thin film, metal deposition needs to be overlapped. The extent of metal spots overlapping can be controlled while thin films form, in order to achieve the desired sheet resistance and surface roughness. The metal overlapping *x* is calculated with the following equation:

(1) $$f_{1}\left(x\right)=\frac{w-\frac{s}{r}}{w}=\left[1-\left(\frac{s}{rw}\right)\right]\times 100\%$$

where *s* denotes the laser scan speed, *r* represents the pulse repetition rate, and *w* represents the radius of the plasma, which is the size of the metal spots on the SCS surface. If *s* > *rw* and *f*_1_(*x*) < 0, there is no plasma overlapping, and LIPAA cannot produce a continuous line width. While *s* = *rw* and *f*_1_(*x*) = 0, metal spots are deposited without overlapping, and a thin metal film with low resistivity cannot be obtained. In general, if 1/(2*rw*) > s > 0, it will produce a decreased line width, and only when *s* << *rw* and *x* > 90% can we have metal spot overlapping for high-quality metal film deposition.

Figure 2 shows the samples fabricated via LIPAA on double-polished c-plane sapphire. The sheet resistance is a function of the metal spot overlapping rate, at a repetition rate of 90 kHz, a target-to-substrate distance of 50 μm, and a metal spot size of 30 μm. The laser scanning speed varies from 0.1 to 30 mm/s, corresponding to a metal overlap rate varying from 99.25 to 99.9%. The sheet resistance is too high and varies little when *x* is between 85 and 95%. An optimal *x* for the lowest sheet resistance is about 99.25%. If *x* is smaller than 99.99%, the sheet resistance is too high to be measured. During LIPAA, both metal material deposition and the removal of deposited materials occur at the same. The removal process happens as the overlapping of metal spots results in absorption of the laser beam by the previously deposited metal spot. At a low *x*, the deposition process dominates and more copper ions are deposited on the surface, and low resistivity can be achieved [21]. However, at a very high metal overlapping rate, the removal process dominates since the deposited metal absorbs the incoming laser pulse energy at the overlap zone, which causes laser ablation to remove the deposited materials. This increases the metal film sheet resistance.

All experiments were carried out along the *z*-axis under the same input laser fluences and incident light wavelengths. The normalized transmittance was plotted against the position of the sample along the *z*-axis. The spectrum of plasma TOF was described as a shifted Maxwell–Boltzmann distribution:

(2) f2(t)=Bt4exp[−(m/2kT0)(Lf−vdt)2t2]

where B is the fitting parameter. m is the mass of ablated plasma of species, which is the ablation depth multiplication product of width. k denotes the Boltzmann constant. *T*_0_ represents the characteristic temperature of the distribution. *L_f_* stands the plasma flight distance from the substrate surface. $v_{d}$ is the center-of-mass velocity that increases more and more slowly, and the velocity then decreases to 0 mm/s. When time is increasing and elapsing, the intensity of plasma reduces more quickly in proportion to the much greater mass. That is to say, the quantity of plasma increasing will reduce the micromachining kinetic energy.

### 3.1. Relation between Laser Fluence and Microstructure Ablation on SCS

**Figure 3:**
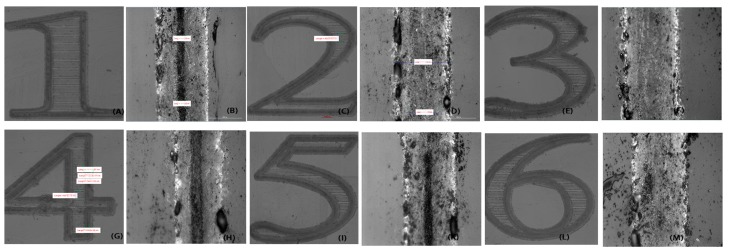
Microscope images of samples ablated with the different laser fluence, respectively: 16.24 J/cm^2^ (**A**,**B**), 16.62 J/cm^2^ (**C**,**D**), 17.0 J/cm^2^ (**E**,**F**), 17.38 J/cm^2^ (**G**,**H**), 17.76 J/cm^2^ (**I**,**K**) and 18.14 J/cm^2^ (**L**,**M**). Substrate-to-target distance = 500 μm; RR = 300 kHz; pulse width = 1 ns; scanning speed = 1 mm/s; *E*_max_ ≅ 5.7 × 10^−5^ J.

**Figure 4:**
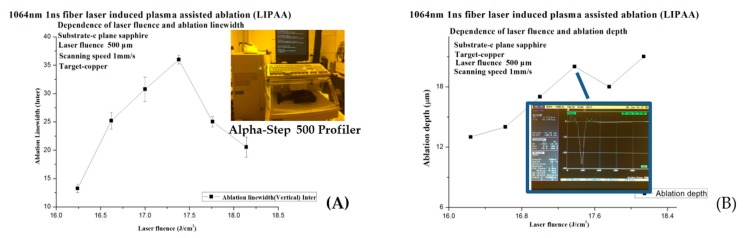
(**A**) The relation between laser fluence and ablation line width, measured with a stylus profilometer (right of picture Figure 4A). (**B**) The deepest cross profile of LIPAA ablation depth measured by Alpha-Step 500 profiler.

As shown in Figure 3 and Figure 4A,B, at the beginning of the LIPAA experiments, the line width increased as laser fluence is increased, and so did the ablation depth because the spectrum of the plasma was increasing at the same time [22,23]. In Equation (2), $v_{d}$ is the increasing plasma kinetic energy. When the fluence further increases to a certain point (approximately 17.5 J/cm^2^), more copper ions excited by the laser beam hit the surface of SCS to ablate the sapphire surface; otherwise, the deposition layer eventually becomes too thick for the laser beam to penetrate and arrive at the sapphire. On the other hand, laser fluence increases will excite the ions and fly in the target direction, precluding the plasma from reaching the sapphire substrate. As a result, small bulk of plasma is involved in the ablation, so both the line width and depth decrease.

### 3.2. Relation between Substrate-to-Target Distance and Microstructure Machining on SCS

**Figure 5:**
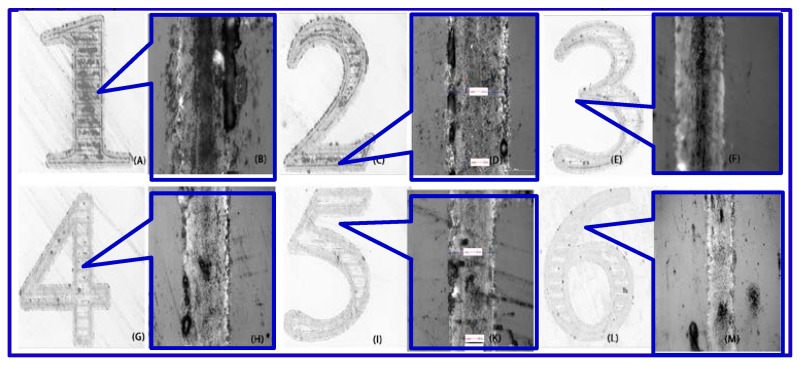
Microscope images of profiles of samples ablated with LIPAA according to different substrate-to-target distances. The distances are as follows: 500 μm ((**A**) 10 times; (**B**) 50 times); 700 μm ((**C**) 10 times; (**D**) 50 times); 900 μm ((**E**) 10 times; (**F**) 50 times); 1100 μm ((**G**) 10 times; (**H**) 50 times); 1300 μm ((**I**) 10 times; (**K**) 50 times); 1500 μm ((**L**) 10 times; (**M**) 50 times). RR = 300 kHz; pulse width = 1 ns; scanning speed = 1 mm/s; laser fluence = 16.24 J/cm^2^); *E*_max_ = 5.7 × 10^−5^ J.

**Figure 6:**
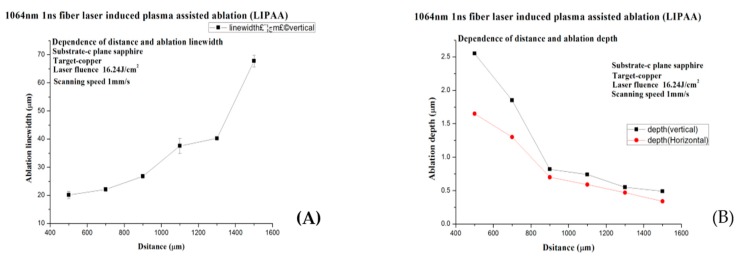
(**A**) The relation between the substrate-to-target distance and the linewidth; (**B**) the depth of the LIPAA for SCS.

As shown in Figure 5 and Figure 6, the laser power is about 10.8 W, the pulse width 5 ns, the repetition rate 90 kHz, and the scanning speed 0.5 mm/s. The substrate-to-target distance is set to 500 μm, 800 μm, 1150 μm, 1200 μm, and 1500 μm, respectively (the metal spot overlap rate is about 99.987%). The time interval of plasma flight increases in proportion to the target-to-substrate distance. The longer distance consumed more kinetic energy for copper plasma species in the plasma flight duration, and the SCS is bombarded by the remaining kinetic energy. Therefore, the ablation line width and depth will reduce if plasma flies a long distance before they reach the SCS backside surface.

As a result, the darkness of the grooves in Figure 5B,D,F,H,K,M became lighter, which indicates that the ablation depth of the grooves decreased when the substrate-to-target distance increased from 500 to 1500 μm. Meanwhile, the ablation line width increased because the plasma expanded during flight and is deposited over a wider area, which led to the formation of a thinner metal film and a higher sheet resistance.

It can be seen from Figure 6A,B that the ablation line width and depth demonstrate some linearity with regard to the substrate-to-target distance. While the distance of the substrate to the target is short, the plasma is collected more together, after that less kinetic energy will be consumed. The line width is dispersed and less deep, and the plasma cannot attain the substrate surface.

### 3.3. The Relation between Scanning Speed and Microstructure Ablation on SCS

**Figure 7:**
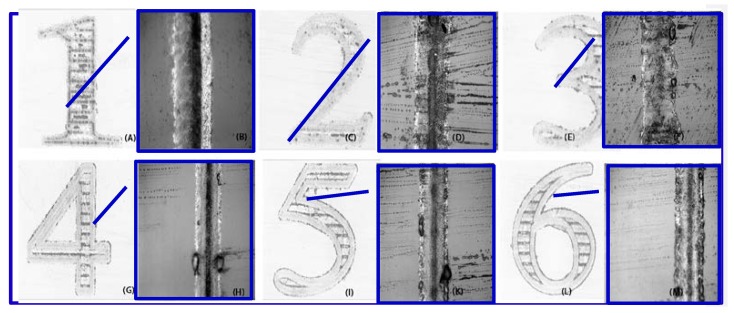
Microscope images of samples ablated with different scanning speeds, respectively: 0.1 mm/s, 0.5 mm/s,1 mm/s, 2 mm/s, 4 mm/s, and 10 mm/s ((**A**,**C**,**E**,**G**,**I**,**L**) 10 times and (**B**,**D**,**F**,**H**,**K**,**M**) 50 times). Substrate-to-target distance = 400 μm; repetition rates = 300 kHz; pulse width = 1 n; laser fluence = 16.24 J/cm^2^. When laser scan speed is low, more plasma overlapped, lower resistant conductive metal ion is deposited.

**Figure 8:**
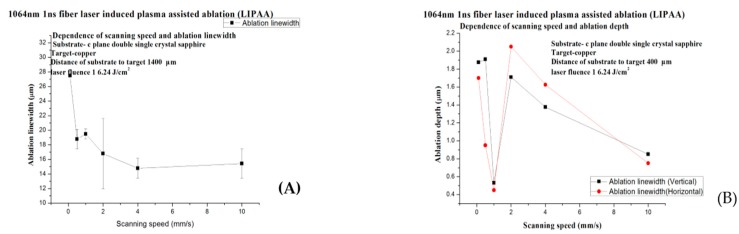
(**A**) The relation between scanning speed and ablation line width, the outer line width. (**B**) The relation between scanning speed and ablation depth.

As shown in Figure 7 and Figure 8, the pulse width is 1 ns, the repetition rate 300 kHz, the substrate-to-target distance 400 μm, and the fluence 16.24 J/cm^2^. The scanning speed is set to 0.1 mm/s (A,B), 0.5 mm/s (C,D), 1 mm/s (E,F), 2 mm/s (G,H), 4 mm/s (I,K), and 10 mm/s (L,M), respectively (overlapping rate 99.987%).

When the laser scans slowly, an enormous laser beam hits the same target area repeatedly, so the ablation line is wider and deeper. When the laser scanning speed increases, the overlapping decreases and fewer copper ions are involved in ablation. Since the kinetic energy does not significantly decrease, the plasma in the lack of binding force is inclined to expand, and the ablation depth does not demonstrate any apparent decrease.

According to Equation (1), the overlapping of laser debris can be controlled. In fact, when the scanning speed is 0 mm/s, the plasma reaches the back of the sapphire; otherwise, the plasma will decrease.

In general, the scanning speed is vital to the ablation quality. When the scanning speed is too low like in Figure 7B, the ablation line is much more continuous than others in Figure 7C,D,F,H,K,M.

### 3.4. The Relation between LIPAA Parameters and Deposition Resistance on S

**Figure 9:**
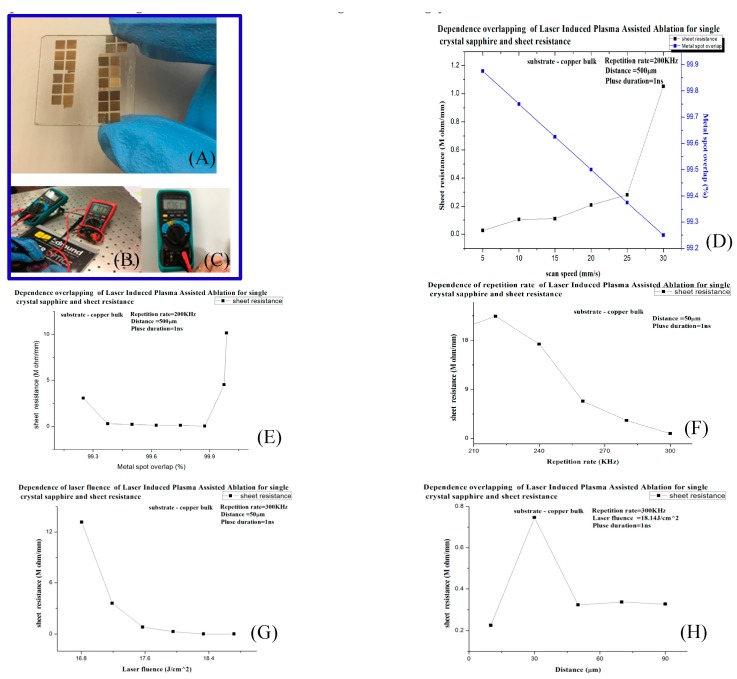
(**A**–**C**) shows SCS metallization samples. (**D**–**F**) The relation between sheet resistance and, respectively, overlapping, scanning speed, and repetition rate. (**G**,**H**) The relation is shown between sheet resistance and, respectively, laser fluence and substrate-to-target distance.

It can see that copper ions are deposited on the surface of SCS, which means the SCS were metallized (Figure 9A–C) and the resistance is measured. When the LIPAA parameters changed, the resistance would change accordingly.

According to the Planckian principle, photon energy will decrease when the repetition rate is lower than a certain value, so the kinetic energy of plasma is not powerful enough to cut and ablate the surface of SCS, but to deposit and make the SCS conductive. The substrate-to-target distance is critical in LIPAA.

As shown in Figure 9D, when scanning speed increases, since the laser overlapping decreases, the amount of excited copper ions decreases. Thus, the number of copper ions deposited on the SCS surface decreases and resistance increases.

Figure 9E shows the trend of resistance when laser overlapping increases. Within a certain range, the increase in overlapping results in a decrease in resistance; however, when the laser overlapping exceeds a certain threshold (approximately 99.99%), the newly excited copper ions may ablate previously deposited ions, and the resistance consequently increases.

In Figure 9F, the metal overlapping rate increases along with the repetition rate. While the repetition rate increases, that is to say, more pulsed laser interacts on target surface and induces more bulk of plasma sticks on surface, above all, according to the formula (1), overlapping rate is increased. At the same time, the resistance on metallization SCS will tune down. While ablation is carried out, the ablation line width will increase.

In Figure 9G, the repetition rate is set to 300 kHz, the pulse duration is 1 ns, and the substrate-to-target distance 50 μm. Since greater quantities of plasma are excited when the copper target is bombarded, resistance decreases when laser fluence increases.

In Figure 9H, an increased substrate-to-target distance within a certain range enables the increase in copper ion deposition and in turn reduces resistance. on the contrary, metal ions will disperse, it means metal bulk will reduce. However, when the flight distance is too long, as distance increases, plasma could not get enough kinetic energy to bombard the rear SCS surface, that is to say, the copper ions cannot easily deposit on the surface of SCS firmly and reduce the resistance very limited.

With so many experiments, the conductive of metallization sapphire will be good at high laser fluence, high repetition rate. On the other hand, the distance factor could not get the ideal resistance rate very often. Of course, the resistance will increase in proportion to the increasing distance of substrate-to-target.

## 4. Conclusions

When the kinetic energy of plasma decreases, the ablation efficiency of metal ions and the line width and depth all decrease together to a certain extent. When the kinetic energy of plasma is lower than the threshold, ablation will not occur, and copper ions will be deposited on the surface of the sapphire. The laser spots and the plasma will reduce. The enhancement of scanning speed and less overlapping will reduce the ablation line width and ablation depth before the ion are enough to click each other, and continue to affect the processing quality. That is to say, the ablation line width and depth will increase in proportion to the laser fluence.

In the process of sapphire metallization, increasing overlapping will greatly help to reduce sapphire sheet resistance. The resistance will be small when laser fluence is high, repetition rate as well, on the contrary, the resistance will get higher while distance is increasing.
